# STAT3 Stabilizes IKKα Protein through Direct Interaction in Transformed and Cancerous Human Breast Epithelial Cells

**DOI:** 10.3390/cancers13010082

**Published:** 2020-12-30

**Authors:** Young-Il Hahn, Soma Saeidi, Su-Jung Kim, Se-Young Park, Na-Young Song, Jie Zheng, Do-Hee Kim, Han-Byoel Lee, Wonshik Han, Dong-Young Noh, Hye-Kyung Na, Young-Joon Surh

**Affiliations:** 1 Department of Molecular Medicine and Biopharmaceutical Sciences, Graduate School of Convergence Science and Technology, Seoul National University, Seoul 08826, Korea; duddlf84@hotmail.com (Y.-I.H.); saeidi@snu.ac.kr (S.S.); 2 Tumor Microenvironment Global Core Research Center, College of Pharmacy, Seoul National University, Seoul 08826, Korea; nynna79@snu.ac.kr (S.-J.K.); jungle0621@naver.com (J.Z.); 3 Department of Oral Biology, Yonsei University College of Dentistry, Seoul 03722, Korea; SYPARK87@yuhs.ac; 4 Department of Chemistry, College of Convergence and Integrated Science, Kyonggi University, Suwon 16227, Korea; calla212@snu.ac.kr; 5 Cancer Research Institute, Seoul National University, Seoul 03080, Korea; hblee.md@snu.ac.kr (H.-B.L.); hanw@snu.ac.kr (W.H.); dynoh@snu.ac.kr (D.-Y.N.); 6 Department of Surgery, Seoul National University College of Medicine, Seoul 03080, Korea; 7 Department of Food Science and Biotechnology, College of Knowledge-Based Services Engineering, Sungshin Women’s University, Seoul 01133, Korea; nhk1228@sungshin.ac.kr

**Keywords:** STAT3, non-canonical NF-κB pathway, IKKα, H-*Ras* MCF-10A cells, breast cancer

## Abstract

**Simple Summary:**

Signal transducer and activator of transcription 3 (STAT3) and nuclear factor-κB (NF-κB) play a cooperative role in inflammation-associated tumorigenesis at multi-levels. The α subunit of the inhibitor of the κB kinase (IKK) complex, IKKα, is involved in both classical and non-classical activation of NF-κB. However, the interplay between STAT3 and IKKα has not been clarified yet. Here, we report overexpression and co-localization of IKKα and STAT3 in human breast cancer tissues as well as in human breast cancer cells, which promotes breast cancer promotion and progression. IKKα was stabilized and upregulated by STAT3. We identified the lysine 44 residue of IKKα as a putative binding site for STAT3. Taken together, these findings propose a novel mechanism responsible for NF-κB activation by STAT3 through stabilization of IKKα. Thus, STAT3 and IKKα could integrate, and coordinately mediate the growth and progression of human breast cancer.

**Abstract:**

Signal transducer and activator of transcription 3 (STAT3) and nuclear factor-κB (NF-κB) are two representative transcription factors that play a critical role in inflammation-associated tumorigenesis through multi-level cooperation. Unlike other types of tumors, breast carcinomas have shown a significant dependency on the non-classical NF-κB pathway as well as the classical one. The α subunit of the inhibitor of the κB kinase (IKK) complex, IKKα, is involved in both classical and non-classical activation of NF-κB. Although the cross-talk between STAT3 and NF-κB has been suggested in several studies, the interplay between STAT3 and the regulators of NF-κB including IKKα has not been fully clarified yet. In this study, we observed overexpression and co-localization of IKKα and STAT3 in human breast cancer tissues as well as in H-*Ras* transformed human breast epithelial (H-*Ras* MCF-10A) and breast cancer (MDA-MB-231) cells. By utilizing small interfering RNA (siRNA) technology, we were able to demonstrate that STAT3 up-regulated IKKα, but not IKKβ or IKKγ, in these cells. This was attributable to direct binding to and subsequent stabilization of IKKα protein by blocking the ubiquitin-proteasome system. Notably, we identified the lysine 44 residue of IKKα as a putative binding site for STAT3. Moreover, siRNA knockdown of *IKKα* attenuated viability, anchorage-independent growth and migratory capabilities of H-*Ras* MCF-10A cells. Taken together, these findings propose a novel mechanism responsible for NF-κB activation by STAT3 through stabilization of IKKα, which contributes to breast cancer promotion and progression. Thus, breaking the STAT3-IKKα alliance can be an alternative therapeutic strategy for the treatment of breast cancer.

## 1. Introduction

Signal transducer and activator of transcription 3 (STAT3) and nuclear factor-κB (NF-κB) are two principal transcription factors that play pivotal roles in inflammation-associated tumorigenesis [1,2,3]. Constitutive activation or overexpression of both transcription factors has been observed in a wide variety of malignancies, which strongly correlates to poor prognostic outcomes in cancer patients [4,5]. Once activated, STAT3 and NF-κB induce expression of genes involved in cell proliferation, transformation, and survival [3,6].

For both STAT3 and NF-κB, phosphorylation is an essential event required for their activation. Upon phosphorylation at the tyrosine 705 residue, STAT3 dimerizes and migrates to the nucleus for transactivation [6]. In the case of NF-κB, two distinct signaling pathways, known as the classical (canonical) and the alternative (non-canonical) pathways, account for its activation. For the classical pathway, phosphorylation of the inhibitor of κB (IκBα) can lead to the release and nuclear localization of the p65/p50 heterodimer, while the phosphorylation and subsequent processing of p100 into p52 are critical for the alternative pathway [7]. The IκB kinase (IKK) complex, composed of IKKα, IKKβ and IKKγ, is involved in directing phosphorylation of IκB. Among these, IKKα is solely responsible for non-classical NF-κB activation through p100 phosphorylation [8].

Considering the shared characteristics in tumorigenesis and phosphorylation-dependent regulation, it is anticipated that STAT3 and NF-κB can interact at multiple levels. STAT3 has been reported to physically interact with p65, a functionally active subunit of NF-κB, thereby retaining the latter transcription factor in the nucleus [9,10]. Activated NF-κB, in turn, up-regulates the pro-inflammatory cytokines, such as interleukin-6 (IL-6), a prototypic STAT3 activator [11], constituting a vicious cycle. Thus, STAT3 and NF-κB sustain the pro-inflammatory and pro-tumorigenic microenvironment in a cooperative manner [12,13].

The multi-level interactions between STAT3 and NF-κB have been elucidated particularly in the context of classical NF-κB activation, while the interplay between STAT3 and non-canonical NF-κB signaling has not been fully addressed. It is noteworthy that both STAT3 and alternative NF-κB pathways are associated with poor prognosis in breast cancer patients [14,15,16]. We have previously reported that inhibition of STAT3 can induce apoptosis in H-*Ras* transformed human breast epithelial (H-*Ras* MCF-10A) cells [17], which corroborates the crucial role of STAT3 in the progression of human breast carcinoma. In the subsequent study, we observed the co-expression of STAT3 and IKKα in human breast carcinoma tissues. This prompted us to more systematically explore the interplay between STAT3 and IKKα and its implications for the growth and progression of breast cancer cells.

## 2. Results

### 2.1. IKKα Is Co-Expressed and Co-Localizes with STAT3 in Human Breast Carcinoma

**Figure 1:**
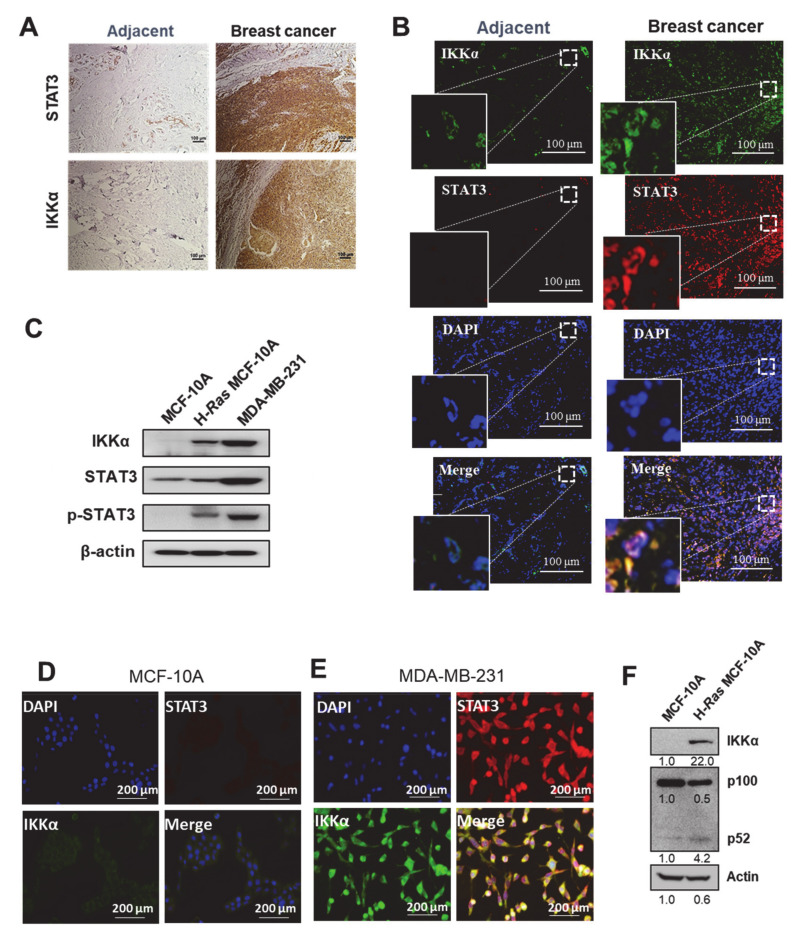
Co-expression of STAT3 and IKKα in human breast carcinoma. (**A**,**B**) Frozen sections from human breast carcinoma and its normal adjacent tissues were stained with antibodies against STAT3 or IKKα. The representative images were obtained by immunohistochemical (**A**) and immunofluorescence (**B**) analyses. 4′,6-Diamidino-2-phenylindole (DAPI), nuclear staining for immunofluorescence. Scale bar, 100 μm. (**C**) Immunoblot analysis shows comparative expression levels of IKKα, phospho-STAT3 (p-STAT3) and STAT3 in MCF-10A, H-*Ras* MCF-10A, and MDA-MB-231 cells. Actin was used as an equal loading control. (**D**,**E**) Immunofluorescence analysis of IKKα and STAT3 in MCF-10A (**D**) and MDA-MB-231 (**E**) cells. DAPI, nuclear staining for immunofluorescence. Scale bar, 200 μm. (**F**) Immunoblot analysis showing comparative expression levels of IKKα, p100, and its cleaved form p52 in MCF-10A and H-*Ras* MCF-10A cells.

We initially investigated the correlation between STAT3 and IKKα, particularly in the context of the non-canonical NF-κB pathway, using specimens from patients with breast carcinoma. As shown in Figure 1A, both IKKα and STAT3 were overexpressed in human breast cancer tissues compared to their normal counterparts. The immunofluorescence staining revealed that IKKα co-expressed and co-localized with STAT3 in tumors of human breast cancer patients (Figure 1B). In conducting mechanistic studies, we initially examined the IKKα expression level in H-*Ras* MCF-10A and human breast cancer MDA-MB-231 cells as well as non-oncogenic MCF-10A cells. Both H-*Ras* MCF-10A and MDA-MB-231 cells had abundant IKKα expression, compared to non-cancerous MCF-10A cells (Figure 1C and Figure S1A). Consistently, immunofluorescence analysis revealed the relatively weak co-expression as well as co-localization of STAT3 and IKKα in MCF-10A cells (Figure 1D), whereas MDA-MB-231 cancer cells exhibited much stronger fluorescence intensity of both proteins (Figure 1E). Contrary to IKKα, the expression of IKKβ and IKKγ, the other subunits of the IKK complex, was marginally increased in H-*Ras* MCF-10A cells (Figure S1B). In line with this observation, the non-canonical NF-κB activation was escalated in IKKα-overexpressing H-*Ras* MCF-10A cells compared to parental MCF-10A cells, as evidenced by increased proteolysis of p100 to form p52 (Figure 1F). These results imply that STAT3 is associated with a non-canonical NF-κB pathway via IKKα. Of note, STAT3 was activated through phosphorylation in H-*Ras* MCF-10A and MDA-MB-231 cells (Figure 1C) as well as human breast cancer tissues (Figure S1C) to a greater extent than their normal counterparts.

### 2.2. IKKα May Promote Human Breast Cancer Progression through Non-Canonical NF-κB Activation

Based on the observation of the co-expression and co-localization of IKKα and STAT3, we speculated that IKKα might also play a role in the progression of human breast carcinoma, as STAT3 does. To test this supposition, a survival analysis was performed by examining the expression levels of the IKK isoforms using the Xena browser dataset for breast cancer. In a breast cancer cohort, the patient group with high IKKα expression exhibited worse overall survival, particularly at the late stage, compared to the low IKKα group (Figure S2A). However, there was no obvious correlation between the overall survival and the expression levels of the other two subunits of the IKK complex (Figure S2B,C). These findings suggest that IKKα overexpression is associated with poor prognosis in breast carcinoma. 

**Figure 2:**
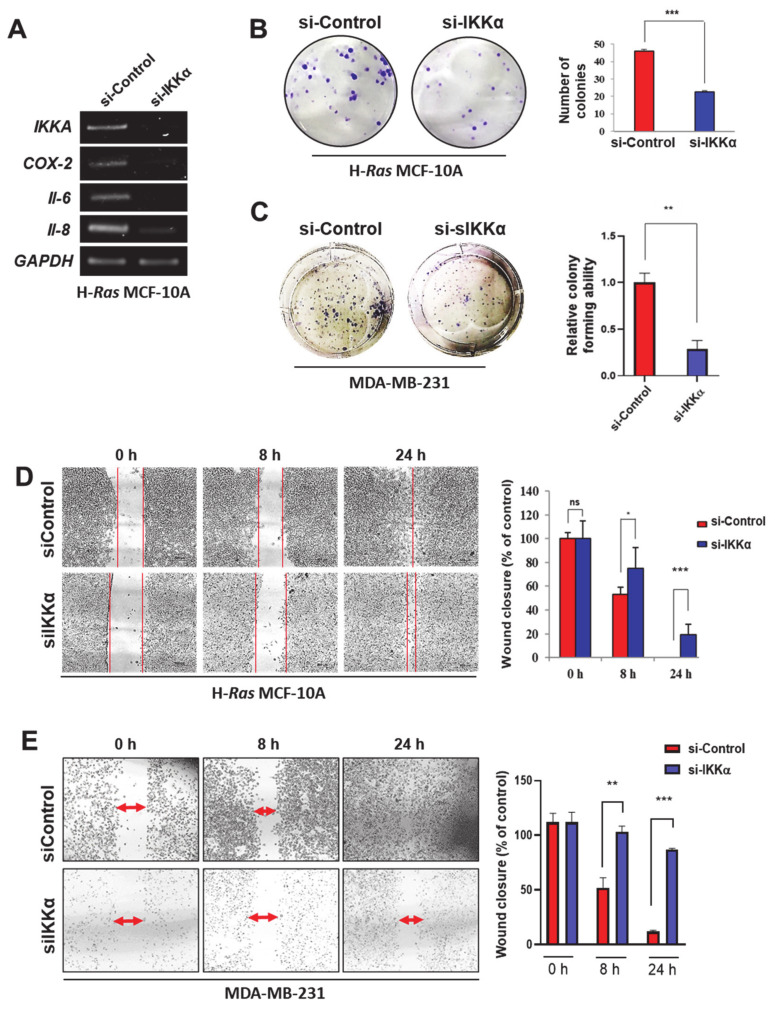
IKKα knockdown suppresses tumorigenesis in H-*Ras* MCF-10A and MDA-MB-231 cells. (**A**) RT-PCR analysis of *IKKα*, *IL-6*, *IL-8*, and *COX-2* mRNA expression in H-*Ras* MCF-10A cells transfected with si-Control or si-IKKα for 48 h. GAPDH, an equal loading control for PCR. (**B**,**C**) H-*Ras* MCF-10A (**B**) and MDA-MB-231 (**C**) cells were plated in a 6-well plate and incubated with si-Control or si-IKKα for 7 days. Then, the colonies were stained with 0.5% crystal violet. The number of colonies was counted and presented as a bar graph. ** *p* < 0.01 (*n* = 3). (**D**,**E**) H-*Ras* MCF-10A (**D**) and MDA-MB-231 (**E**) cells were seeded in an ibidi culture insert and then transfected with si-Control or si-IKKα for 48 h. The progression of cell migration was visualized under the microscope and presented as a bar graph. ns, not significant; * *p* < 0.05; ** *p* < 0.01; *** *p* < 0.001 (*n* = 3).

To further assess the role of IKKα in the progression of breast carcinoma, the gene silencing strategy using a siRNA against *IKKα* was adopted. As depicted in Figure 2A, *IKKα* knockdown reduced the expression of representative pro-inflammatory genes (*COX-2*, *IL-6*, and *IL-8*), which is under the control of both STAT3 and NF-κB [18,19], in H-*Ras* MCF-10A cells. Furthermore, the siRNA knockdown of *IKKα* markedly suppressed the colony forming (Figure 2B,C) and migratory (Figure 2D,E) abilities of H-*Ras* MCF-10A and MDA-MB-231 cells, respectively. These data indicate that IKKα exerts pro-oncogenic functions by stimulating the alternative NF-κB pathway in breast cancer.

### 2.3. STAT3 Regulates IKKα Expression in H-Ras Transformed and Cancerous Human Breast Epithelial Cell Lines

**Figure 3:**
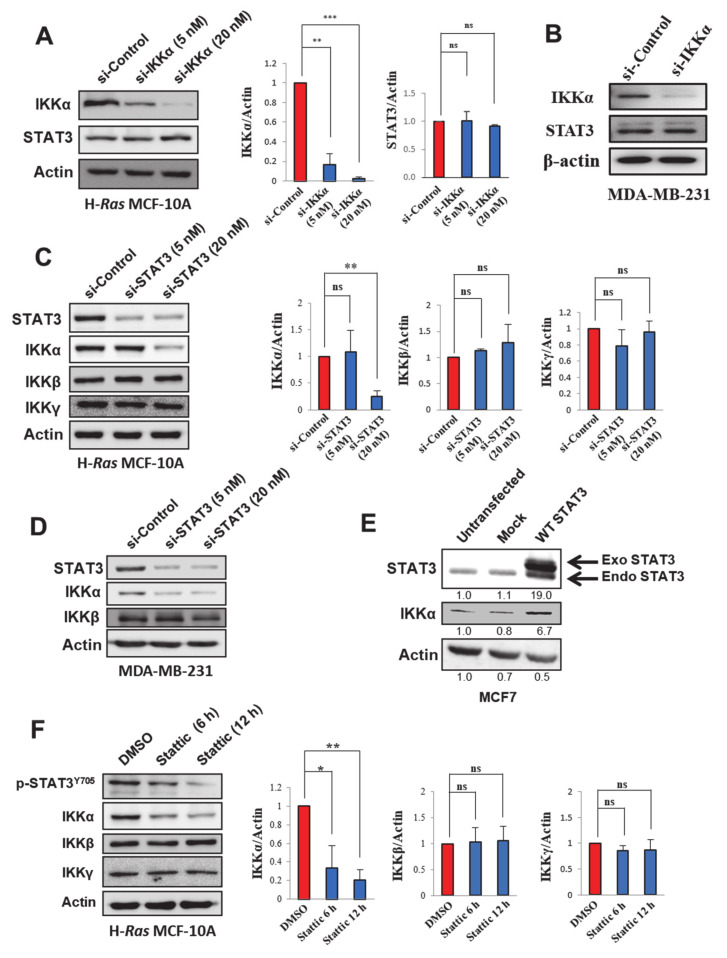
STAT3-dependent expression of IKKα in human breast cancer cell lines. (**A**,**B**) Immunoblot analysis of IKKα and STAT3 in H-*Ras* MCF-10A (**A**) and MDA-MB-231 (**B**). Cells were transfected with si-Control or si-IKKα for 48 h, and expression of STAT3 was measured by immunoblot analysis. Actin, a loading control for immunoblotting. For Figure 3A, the band intensities were normalized by the actin level and presented as bar graphs. ns, not significant; ** *p* < 0.01; *** *p* < 0.001 (*n* = 3). (**C**,**D**) Immunoblot analysis of STAT3, IKKα, IKKβ, and/or IKKγ expression levels in H-*Ras* MCF-10A (**C**) and MDA-MB-231 (**D**) cells transfected with si-Control or si-STAT3 (5 and 20 nM) for 48 h. For Figure 3C, the band intensities were normalized by the actin level and presented as bar graphs. ns, not significant; ** *p* < 0.01 (*n* = 3). (**E**) Immunoblot analysis of IKKα and STAT3 in MCF7 cells transfected with mock or STAT3-overexpressing vectors. (**F**) The H-*Ras* MCF-10A cells were treated with Stattic (1 µM) for 6 or 12 h. The expression levels of p-STAT3, IKKα, IKKβ, and IKKγ were assessed by immunoblot analysis. The band intensities were normalized by the actin level and presented as bar graphs. ns, not significant; * *p* < 0.05; ** *p* < 0.01 (*n* = 3).

The co-expression and coordinated oncogenic functions of IKKα and STAT3 imply a cross-talk between these two signaling proteins in human breast carcinoma. In order to clarify this implication, cells were transfected with a siRNA against either *IKKα* or *STAT3*. The silencing of *IKKα* was not able to alter the expression of STAT3 in H-*Ras* MCF-10A (Figure 3A), MDA-MB-231 (Figure 3B), and MCF-7 (Figure S3A) cells. However, *STAT3* knockdown significantly reduced the expression of IKKα in H-*Ras* MCF-10A (Figure 3C and Figure S3B), MDA-MB-231 (Figure 3D), and MCF7 (Figure S3A) cells. On the contrary, the ectopic expression of STAT3 elevated the IKKα expression level in human breast cancer MCF7 cells (Figure 3E), lending further support to the notion that STAT3 up-regulates IKKα expression. In addition to siRNA directed to silence *STAT3*, treatment with Stattic, a small molecule known to selectively inhibit the STAT3 activation [20], down-regulated IKKα protein expression (Figure 3F) without altering *IKKα* mRNA expression (Figure S3C). These findings suggest that not only STAT3 expression, but also its activity is important for IKKα regulation. Notably, neither genetic nor pharmacologic inhibition of STAT3 changed the expression levels of IKKβ and IKKγ (Figure 3C,D,F). However, *STAT3* silencing blocked proteolysis of p100, a marker of alternative NF-κB activation, in H-*Ras* MCF-10A cells (Figure S3D), further supporting the role for STAT3 in IKKα-mediated non-canonical NF-κB activation. Taken together, these results suggest that STAT3 specifically up-regulates the protein expression of the α subunit in the IKK complex, a key player in the non-canonical NF-κB pathway.

### 2.4. STAT3 Stabilizes IKKα Protein in H-Ras MCF-10A Cells

**Figure 4:**
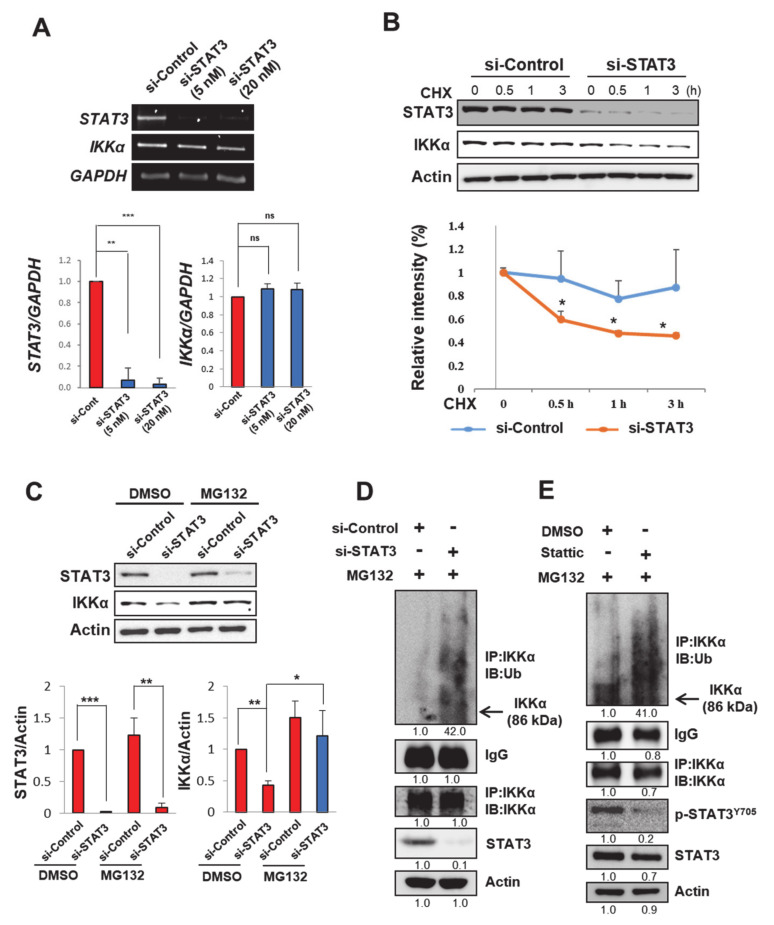
STAT3 protects IKKα from ubiquitin-dependent proteasomal degradation in H-*Ras* MCF-10A cells. (**A**) RT-PCR analysis of STAT3 and IKKα mRNA expression in H-*Ras* MCF-10A cells transfected with si-Control or si-STAT3 (5 and 20 nM) for 48 h. The bar graphs show the relative intensities of *STAT3* (bottom panel, left) and *IKKα* (bottom panel, right) measured by densitometry analyses. *GAPDH*, an equal loading control for PCR. ns, not significant; ** *p* < 0.01; *** *p* < 0.001 (*n* = 3). (**B**) The H-*Ras* MCF-10A cells were incubated with si-Control or si-STAT3 for 24 h, followed by treatment with CHX (20 μg/mL) for various time points. The expression of STAT3 and IKKα was analyzed by immunoblot analysis (upper) and the relative intensity of IKKα was measured and plotted over time (bottom). * *p* < 0.05 (*n* = 3). (**C**) H-*Ras* MCF-10A cells were pre-incubated with si-Control or si-STAT3 for 48 h and then treated with MG132 (10 μM) for an additional 2 h. The expression levels of STAT3 and IKKα were analyzed by immunoblot analysis (upper), and the band intensities were normalized and presented as a bar graph (bottom). * *p* < 0.05; ** *p* < 0.01; *** *p* < 0.001 (*n* = 3). (**D**) Immunoprecipitation analysis showing ubiquitination of IKKα. H-*Ras* MCF-10A cells treated with MG132 for 2 h after pre-incubation with si-Control or si-STAT3 for 48 h. Immunoprecipitation was performed with an IKKα antibody and then the immunocomplexes were gel-separated and blotted with IKKα or ubiquitin antibodies. (**E**) Immunoblot analysis of IKKα expression and ubiquitination in H-*Ras* MCF-10A cells exposed to MG132 for 2 h post-treatment with or without Stattic (1 µM) for 48 h. Immunoprecipitation was performed with an IKKα antibody, followed by immunoblotting with a ubiquitin or an IKKα antibody.

Next, we investigated how STAT3 up-regulates IKKα expression. While both siRNA knockdown (Figure 3C) and pharmacologic inhibition (Figure 3F) of STAT3 reduced the expression of IKKα protein, there was no alteration in the expression levels of its mRNA transcript in H-*Ras* MCF-10A cells (Figure 4A and Figure S3C). Furthermore, the cyclohexamide (CHX) chase assay showed that the half-life of the IKKα protein was markedly declined by STAT3 silencing in H-*Ras* MCF-10A cells (Figure 4B). Thus, it is likely that STAT3 stabilizes IKKα protein, rather than promoting its de novo synthesis. In support of this assumption, a proteasome inhibitor MG132 fully restored IKKα protein expression, which was abolished by *STAT3* knockdown (Figure 4C). However, blocking either the expression (Figure 4D) or activity (Figure 4E) of STAT3 enhanced the poly-ubiquitination of IKKα in H-*Ras* MCF-10A cells. Thus, STAT3 protects IKKα from the ubiquitin-dependent proteasomal degradation, resulting in the stabilization and accumulation of IKKα.

### 2.5. STAT3 Physically Interacts with IKKα, Presumably at Lys44

**Figure 5:**
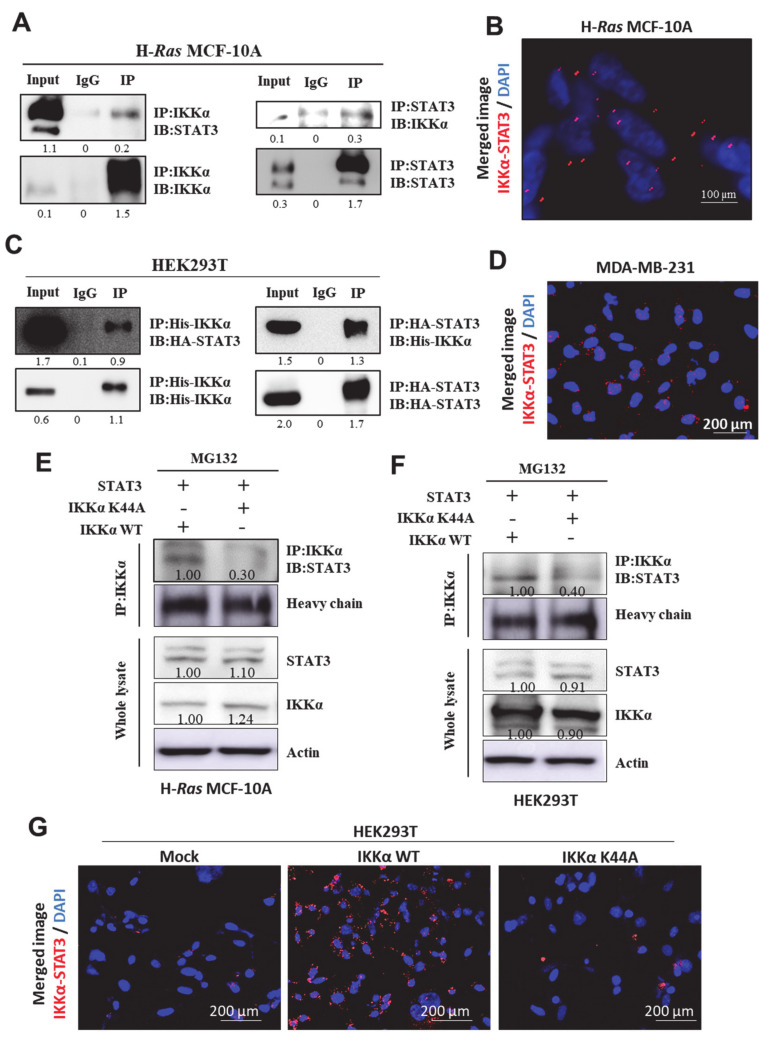
STAT3 directly interacts with IKKα in vitro. (**A**,**C**) IP analysis showing a physical interaction between IKKα and STAT3 in H-*Ras* MCF-10A (**A**) and HEK293T (**C**) cells. Immunoprecipitation was performed with IKKα (left) or STAT3 (right) antibodies, followed by immunoblotting with antibodies for STAT3 and IKKα, respectively. IgG, a negative control for immunoprecipitation; input, total protein lysate. (**B**,**D**) Duolink analysis showing the interaction between STAT3 and IKKα in H-*Ras* MCF-10A (**B**) and MDA-MB-231 (**D**) cells. The representative image was visualized under the fluorescent microscope. (**E**,**F**) H-*Ras* MCF-10A (**E**) and HEK293T (**F**) cells were transfected with IKKα wild type (WT) or K44A mutant (K44A) in the presence of STAT3 construct for 48 h, followed by MG132 treatment for additional 2 h. Immunoprecipitation analysis showing the interaction between STAT3 and either WT or K44A IKKα. (**G**) Duolink analysis showing the STAT3 binding to IKKα WT or K44A mutant in HEK293T cells.

It has been reported that IKKα protein stability is enhanced through physical interaction with other proteins, such as Hsp90 and Cdc37 [21,22]. As IKKα and STAT3 co-localized in human breast cancer patient tissues as well as human breast cancer cell lines (Figure 1C,E,F), we speculated that STAT3 could stabilize IKKα protein through physical interaction. The direct interaction between STAT3 and IKKα was evident in H-*Ras* MCF-10A cells, which was revealed by co-immunoprecipitation (Figure 5A) and an in situ proximity ligation assay (PLA) (Figure 5B). The exogenous interaction between STAT3 and IKKα was also assessed in HEK293T (Figure 5C) and MCF-10A (Figure S4A) cells after ectopic expression of both proteins. The physical interaction between STAT3 and IKKα was also confirmed in MDA-MB-231 cells by PLA (Figure 5D). Again, STAT3 was not able to directly interact with IKKβ and IKKγ (Figure S4B). These data clearly show that STAT3 specifically binds to the α subunit of IKK complex, other than β or γ subunits. 

Previously, Xiao et al. have demonstrated that the lysine 44 residue within the kinase domain of IKKα is crucial for its protein stability [23]. Thus, we speculated that STAT3 might physically bind to the lysine 44 of IKKα protein to prevent its degradation. Mutation of lysine 44 to alanine (K44A) on IKKα interrupted its interaction with STAT3 in both H-*Ras* MCF-10A (Figure 5E) and HEK293T (Figure 5F and Figure S4C) cells. The PLA assay further confirmed that K44A mutation in IKKα abolished its interaction with STAT3 in HEK293T cells (Figure 5G). All these data, taken together, support that STAT3 directly binds to IKKα, particularly at lysine 44, and thereby enhances its protein stability by blocking the ubiquitin-dependent proteasomal degradation. This STAT3-dependent IKKα stabilization can further escalate tumorigenesis in human breast carcinoma through the non-canonical activation of NF-κB.

## 3. Discussion

STAT3 and NF-κB are the major transcription factors known to regulate the expression of genes involved in immune responses, cell proliferation, and survival, linking inflammation and carcinogenesis [24]. Constitutive activation of STAT3 or NF-κB up-regulates major pro-inflammatory cytokines, such as TNFα, IL-1, and IL-6, which can promote cancer cell proliferation, angiogenesis, and metastasis [2,25,26]. Furthermore, STAT3 conditional knockout mice were found to be less susceptible to chemically induced skin [27] and colon [28] carcinogenesis. Similarly, either IKKβ ablation or p65 deficiency suppresses tumorigenesis in these mouse models, implying that STAT3 and NF-κB phenocopy each other peculiarly in the context of promoting tumorigenesis [12,29,30]. 

By examining the data in the catalogue of somatic mutations in cancer (COSMIC), Kaltschmidt et al. have found that IKKα is highly up-regulated in human breast cancer tissues, compared to other types of cancers [31]. This implies that in mammary tumors, the non-classical NF-κB pathway is more likely to play a principal role in the oncogenic signal transduction than the classical pathway. In agreement with this notion, up-regulation of either IKKα or p100 is associated with poor survival in the breast cancer patients [14,16]. We also observed that the expression of IKKα, rather than IKKβ or IKKγ, was positively correlated with poor prognosis, according to the Xena browser in TCGA BRCA database (Figure S2). Moreover, it has been reported that the mammary gland-specific p100 overexpression induces the up-regulation of the pro-tumorigenic proteins in mice [32]. With regard to uncovering the novel therapeutic targets and/or strategies to treat breast carcinomas, it would be worthwhile to understand how STAT3 interacts with the non-canonical NF-κB pathway. 

STAT3 and NF-κB collaborate at several different levels. Some of the pro-inflammatory genes, such as *COX-2* and *IL-6*, contain the binding sites for both STAT3 and NF-κB in their promoter regions, allowing an interdependent regulation [12,13]. Moreover, STAT3 has been reported to physically interact with RelA/p65 and p50 subunits of NF-κB, particularly responsible for the classical activation of NF-κB [9,10,33]. In addition to the direct binding, STAT3 can mediate sustained nuclear localization of RelA/p65, resulting in a prolonged activation of the canonical NF-κB signaling [9]. However, how STAT3 interplays with the non-canonical NF-κB pathway has remained elusive, although the alternative NF-κB activation also takes an important role in the inflammation and tumorigenesis. 

**Figure 6:**
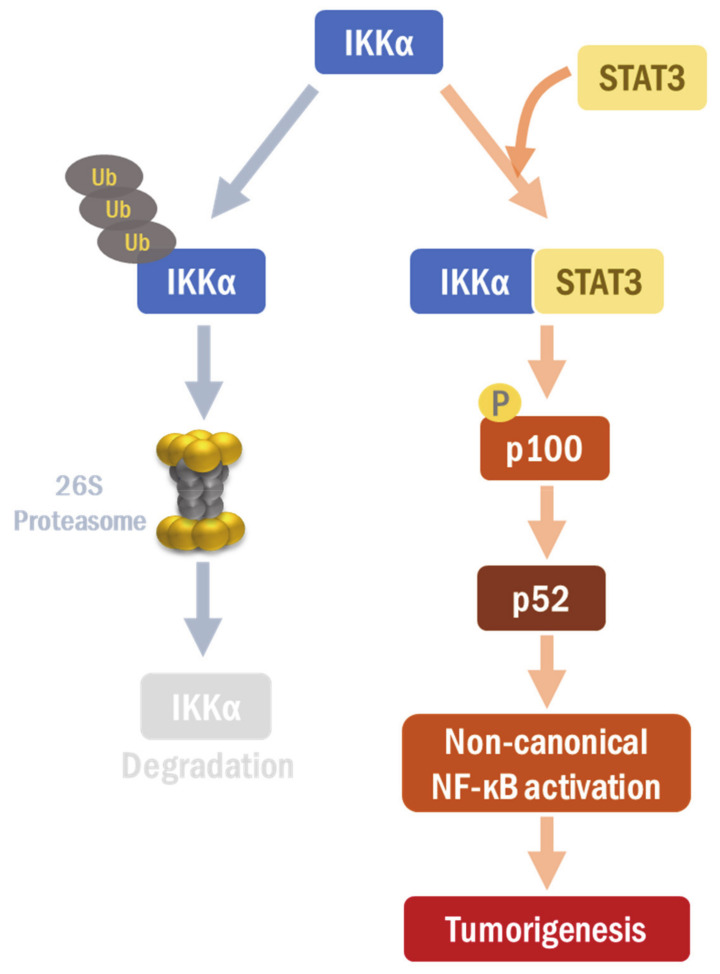
A proposed scheme of the interaction between STAT3 and IKKα. STAT3 directly interacts with and stabilizes IKKα, thereby regulating non-canonical NF-κB signaling.

In the current study, STAT3 was found to regulate the protein expression of IKKα, but not that of IKKβ or IKKγ, in H-*Ras* MCF-10A cells and MDA-MB-231 breast cancer cells. The silencing of IKKα remarkably suppressed proliferative and migratory abilities of these cells. While IKKβ and γ do not play much of a role in the non-classical activation, IKKα is well known to be involved in both classical and non-classical NF-κB pathways [34]. In our study, IKKα triggered the non-canonical NF-κB activation in H-*Ras* MCF-10A cells in which STAT3 signaling is overactivated. Thus, our findings strongly support that STAT3 is closely inter-connected with the alternative NF-κB pathway via IKKα regulation. We also noticed that STAT3 directly interacted with IKKα and stabilized it by inhibiting the ubiquitin-dependent proteasomal degradation (Figure 6). Xiao et al. have shown that IKKα mutation on K44A facilitates its protein degradation, indicating that the lysine 44 is important to maintain IKKα protein stability [23]. Interestingly, we found that STAT3 failed to form a complex with the IKKα K44A mutant. This may explain why the IKKα K44A mutant is vulnerable to degradation. The lysine 44 of the IKKα protein provides a contact point to STAT3, which contributes to the stabilization of IKKα. Further investigation is required to fully elucidate the underlying mechanism. 

## 4. Materials and Methods

### 4.1. Tissues, Cell Culture, and siRNA Knockdown of Gene Expression

Human breast cancer tissue slides (including both adjacent and malignant tissues; a total of three pairs) were obtained from the biorepository of Lab of Breast Cancer Biology at the Cancer Research Institute, Seoul National University. Non-transformed human mammary epithelial MCF-10A (ATCC: CRL-10317) cells and H-*Ras* MCF-10A (obtained from Prof. Aree Moon of Duksung Women’s University) cells were grown in DMEM/F-12 supplemented with 5% horse serum, 100 ng/mL cholera toxin, 20 ng/mL human epidermal growth factor, 10 μg/mL insulin, 0.5 μg/mL hydrocortisone, 2 mM L-glutamine, and 100 units/mL penicillin/streptomycin. Human embryonic kidney HEK293T cells (ATCC: CRL-11268) and human breast cancer MDA-MB-231 (ATCC: HTB-26) and MCF-7 (ATCC: HTB-22) cell lines were maintained in DMEM and RPMI 1640 supplemented with 10% fetal bovine serum and 100 units/mL penicillin/streptomycin. For gene silencing experiments, cells were transfected with siRNA for human *STAT3* (target sequence 5′-CUAUCUAAGCCCUAGGUUUdTdT-3′ and 5′-CCUAGGGCUUAGAUAGdTdT-3′), human *IKKα* (target sequences 5′-GAAGAAAUGGCUAUGAACAdTdT-3′ and 5′-UGUUCAUAGCCAUUUCUUCdTdT-3′), or scrambled negative control (target sequence 5′- CCUACGCCACCAAUUUCGU-3′, and 5′-ACGAAAUUGGUGGCGUAGG-3′) using the Lipofectamine RNAi-MAX transfection reagents according to the instructions supplied by the manufacturer (Invitrogen; Carlsbad, CA, USA). Each siRNA was diluted in serum-free media in a final volume of 6 μL. Transfection reagents diluted in serum-free media in a final volume of 10 μL were added to each well. After a 30-min incubation at room temperature, cells were added to the plates. After 48-h transfection, cells were lysed for further analysis. We custom prepared those siRNAs from Bioneer (Seoul, Korea). For ectopic expression of STAT3 and IKKα, cells were transfected with STAT3 overexpression vector pCMV-HA-STAT3 plus pcDNA3-IKKa WT or mutant (K44A) plasmid.

### 4.2. Reagents and Antibodies

Recombinant human IL-6 and MG132 were purchased from R&D Systems, Inc. (Minneapolis, MN, USA). CHX was purchased from Sigma Aldrich (St. Louis, MO, USA). Primary antibodies for p-STAT3^Y705^, STAT3, IKKα, IKKβ, IKKγ, p100, HA-tag (anti-rabbit), and His-tag (rabbit) were purchased from Cell Signaling Technology (Danvers, MA, USA). Primary antibody against His-tag (mouse) was purchased from Santa Cruz Biotech (Santa Cruz, CA, USA). An anti-ubiquitin antibody was obtained from Thermo Fisher Scientific (Waltham, MA, USA). DAPI was purchased from Invitrogen. 

### 4.3. Public Data Resources and Survival Analysis

The Kaplan-Meier plots of the five-year overall survival were generated by the USCS Xena browser (https://xenabrowser.net) in TCGA BRCA database. The patients were divided into high and low IKKα/IKKβ/IKKγ gene expression groups according to median expression. 

### 4.4. Immunoblot and Immunoprecipitaion Assays

For immunoblot analysis, cell lysates were heated for 5 min at 95 °C in the protein gel-loading buffer, and applied to 7–12% sodium dodecyl sulfate-polyacrylamide gels. The separated proteins were transferred to a PVDF membrane, blocked in 5% nonfat milk, and analyzed using the specific primary antibodies. Proteins were detected using a chemiluminescence reagent. For immunoprecipitation, cell lysates were first treated with STAT3 antibody or IKKα antibody at 4 °C overnight using a rotator, followed by incubation with protein A/G beads (Santa Cruz Biotech). The immunoprecipitates were washed with lysis buffer three times and subjected to IB with indicated antibodies. The band intensity was measured using Gel-Pro Analyzer™ software and normalized with the loading control. All original blot images were shown in Figures S5–S13. 

### 4.5. Immunohistochemistry and Immunofluorescence Staining

Paraffin-embedded tissues were deparaffinized with xylene. For antigen retrieval, tissues were heated in a microwave oven with citrate buffer (DakoCytomation; Glostrup, Denmark). The tissue sections were incubated with 0.2% Triton X-100 for permeabilization, then blocked with 3% bovine serum albumin (BSA) in phosphate-buffered saline (PBS) for 1 h. Then, the sections were incubated overnight at 4 °C with specific primary antibodies, followed by incubation with secondary antibodies conjugated with horse radish peroxidase for immunohistochemistry, or conjugated with fluorophore for immunofluorescence staining. For immunofluorescence staining, H-*Ras* MCF-10A cells were cultured (2 × 10^4^ in an eight-chamber slide), fixed with 95% methanol/5% acetic acid for 10 min and permeabilized with 0.2% Triton X-100 for 5 min. The cells were then blocked with 5% BSA in PBS with Tween 20 (PBS-T) for 1 h and incubated with specific primary antibodies in PBS-T containing 1% BSA at 1:100 dilution at 4 °C overnight, followed by staining with Alexa Fluor secondary antibodies (Invitrogen). Finally, the slides were mounted with DAPI (Invitrogen) and visualized under a florescent microscope. 

### 4.6. Reverse Transcription Polymerase Chain Reaction (RT-PCR)

Total RNA was isolated using TRIzol reagent (Invitrogen). The cDNA was synthesized from the isolated RNA with a reverse transcriptase (Promega; Madison, WI, USA), according to the manufacturer’s instruction. For PCR, primers included the following: *IKKα*, 5′- AGTTCTTCAGGATGTTGGTGG-3′ and 5′-CCAGACACATAGTGCACTGCT-3′; *STAT3*, 5′- AGAATCACGCCTTCTACAGACTG-3′ and 5′-ACGATTCTCTCCTCCAGCATC-3′; *COX-2*, 5′-GCTGAGCCATACAGCAAATCC-3′ and 5′-GGGAGTCGGGCAATCATCAG-3′; *IL-6*, 5′-GTGTGAAAGCAGCAAAGAGGC-3′ and 5′-CTGGAGGTACTCTAGGTATAC-3′; and *IL-8*, 5′-ATGACTTCCAAGCTGGCCGTGGCT-3′ and 5′-TCTCAGCCCT CTTCAAAAACTTCT-3′.

### 4.7. Soft Agar Colony Forming and Migration Assays

For the soft agar colony forming assay, cells (1 × 10^5^) were added to media containing 3.3% agarose and layered onto a 60 mm^2^-dish containing 0.5% agar in DMEM/F12 media. The cells were incubated with indicated siRNAs for 7 days. Once a colony formed, the colonies were fixed in cold methanol and stained with crystal violet. The colonies were visualized under a microscope (Nikon) and quantified. For the migration assay, cells were seeded onto Culture-Inserts^®^ (ibidi; Regensburg, Germany) to generate a wound gap. After incubation with indicated siRNAs for 8 or 24 h, the insert was gently removed. The progression of wound closure was visualized at various time points under the microscope. 

### 4.8. CHX Chase Assay

H-*Ras* MCF10A cells were transfected with scrambled or *STAT3* siRNAs. At 24 h post-transfection, cells were treated with CHX (20 µg/mL) at various time points from 0 to 3 h. Then, the cells were harvested and lysed. The cell lysates were subjected to immunoblot analysis with STAT3 and IKKα antibodies to determine the half-life of IKKα protein. 

### 4.9. PLA

PLA was performed using the DUOLink^TM^ kit (OLINK; Uppsala, Sweden). H-*Ras* MCF-10A and MDA-MB-231 cells were seeded onto the glass slide and incubated for 24 h. After incubation, the cells were washed with PBS, fixed with 95% methanol/5% acetic acid for 10 min, permeabilized with 0.2% Triton X-100 for 5 min and again washed with PBS three times. Cells were then blocked with 5% BSA in PBS-T for 1 h and incubated with anti-STAT3 and anti-IKKα in PBS-T containing 1% BSA at 1:100 dilution at 4 °C overnight. After incubation, the cells were washed with PBS-T and then incubated with PLA plus and minus affinity probes for 1 h at 37 °C. The probes were then hybridized with a ligase. The DNA was amplified and detected under the fluorescence microscopy. 

### 4.10. Statistical Analysis

All experiments were performed in triplicate and repeated three times. Difference between groups was analyzed by Student’s unpaired *t*-test. The data were presented as means ± standard deviations (S.D.) of at least three independent repeats. The values of *p* less than 0.05 were considered to indicate statistical significance. 

## 5. Conclusions

STAT3 stabilizes and up-regulates IKKα expression, responsible for the non-canonical NF-κB activation, in H-*Ras* MCF-10A cells, exacerbating tumorigenesis (Figure 6). The STAT3-IKKα interaction plays a crucial role for IKKα stabilization, which can provide a novel therapeutic target for breast cancer treatment. Thus, STAT3 could integrate between both distinct NF-κB signaling pathways, which coordinately mediates the inflammation-associated carcinogenesis in human breast carcinomas. However, further studies will be necessary to clarify the molecular and cellular contexts in which STAT3 determines its oncogenic partner between canonical and non-canonical NF-κB pathways.

## Data Availability

All the data presented in this study are included in this article and its supplementary material file.

## Supplementary Materials

The following materials are available online at https://www.mdpi.com/2072-6694/13/1/82/s1. Figure S1: Protein expression levels of IKK isoforms and pSTAT3 in human breast carcinoma tissues and cell lines; Figure S2: Survival analyses based on the expression of IKK isoforms in human breast cancer cohort; Figure S3: STAT3-dependent expression of IKKα in human breast cancer cell lines; Figure S4: STAT3 is not able to interact with other IKK subunits; Figure S5: The original blot images of Figure 1; Figure S6: The original blot images of Figure 2; Figure S7: The original blot images of Figure 3A,B; Figure S8: The original blot images of Figure 3C,D; Figure S9: The original blot images of Figure 3E,F; Figure S10: The original blot images of Figure 4A,B; Figure S11: The original blot images of Figure 4C,D; Figure S12: The original blot images of Figure 5; Figure S13: The original blot images of Figures S1 and S3.
