# Supplementary material for: STAT3 Stabilizes IKKα Protein through Direct Interaction in Transformed and Cancerous Human Breast Epithelial Cells
Source: Cancers (Basel). 2020 Dec 30;13(1):82. doi: 10.3390/cancers13010082 (PMC7795115; doi:10.3390/cancers13010082)
Supplement: Supplementary file 1 [file cancers-13-00082-s001.pdf]

cancers

MDPI

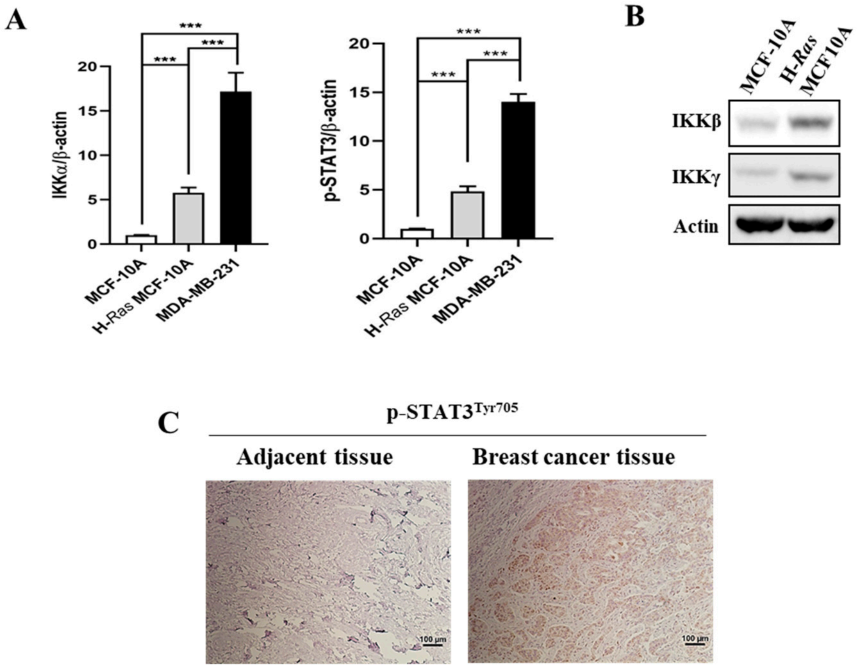

Figure S1. Protein expression levels of IKK isoforms and p-STAT3 in human breast carcinoma tissues and cell lines (A) Quntification of data (Fig. 1C) on IKKα and p-STAT3 protein expression in H-*Ras* MCF-10A and MDA-MB231 cells and normal counterpart MCF-10A cells. Actin, a protein loading control. **(B)** IB analysis of IKKβ and IKKγ in H-*Ras* MCF-10A and normal counterpart MCF-10A cells. **(C)** IHC analysis of comparative expression of p-STAT3 between adjacent normal and tumor tissues of human breast cancer patients.

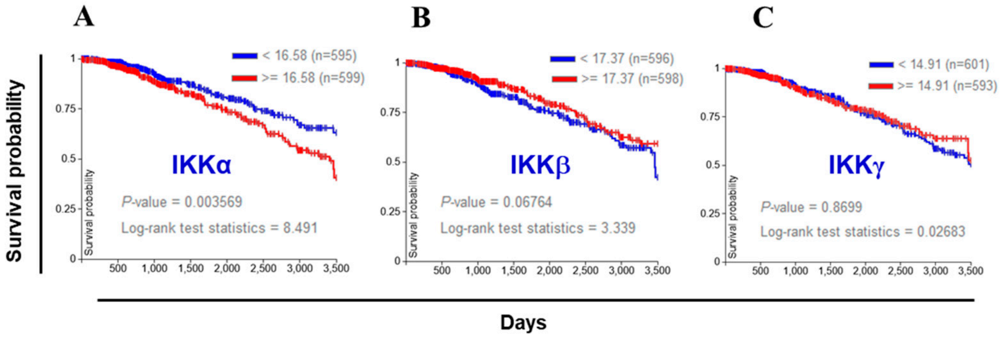

Figure S2. Survival analyses based on expression of IKK isoforms in human breast cancer cohort (A-C) The Kaplan-Meier plots showing 5-year overall survival of breast cancer patients with low and high expression of IKK isoforms in TCGA Breast Cancer (TCGA BRCA). The survival curves were generated by using UCSC Xena (https://xenabrowser.net).

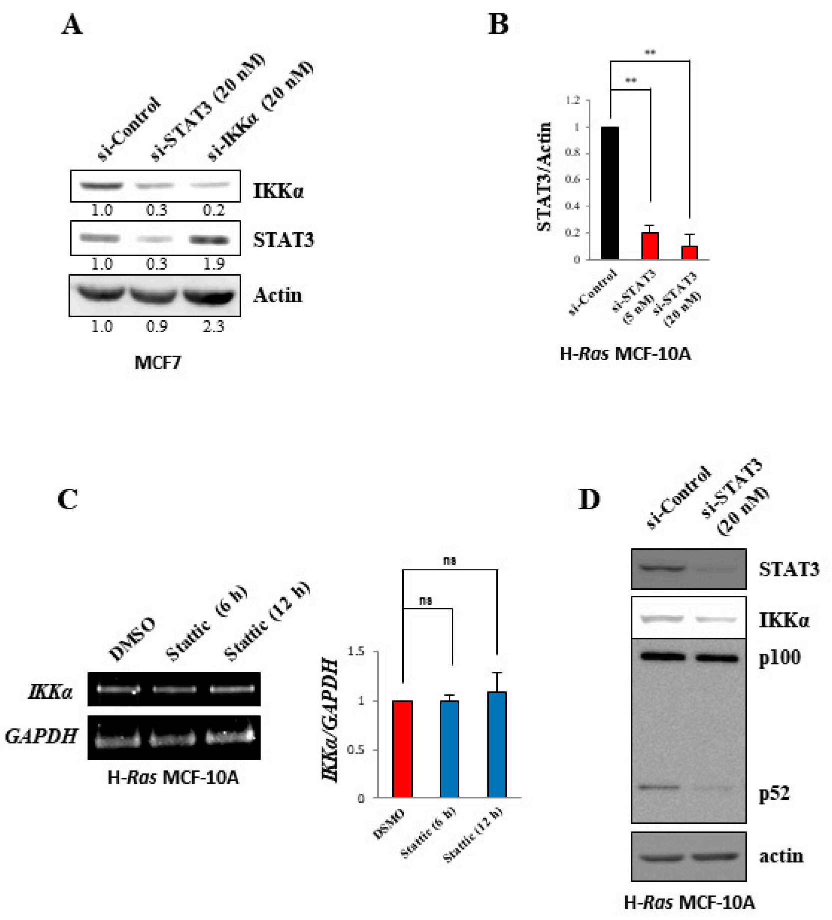

Figure S3. STAT3-dependent expression of IKKα in human breast cancer cell lines (A) IB analysis of IKKα and STAT3 in MCF7 cells transfected with si-Control, si-STAT3 (20 nM), or si-IKKα (20 nM) for 48 h. **(B)** The densitometry calculations for IB data shown in Figure 3C. The band intensities were normalized by the actin level and presented as bar graphs. **, $p < 0.005$. **(B)** IB analysis of STAT3, IKKα and p100 proteolysis in H-*Ras* MCF-10A cells transfected with si-Cont or si-STAT3 (20 nM) for 48 h. **(C)** RT-PCR analysis of *IKKα* mRNA expression in H-*Ras* MCF-10A cells exposed to Stattic (1 μM) for indicated time periods (left). The band intensities were normalized and presented as a bar graph (right). *GAPDH*, a loading control. ns, not significant. **(D)** IB analysis showing comparative expression levels of STAT3, IKKα, p100 and its cleaved form p52 in H-*Ras* MCF-10A cells transfected with si-Control or si-STAT3 (20 nM).

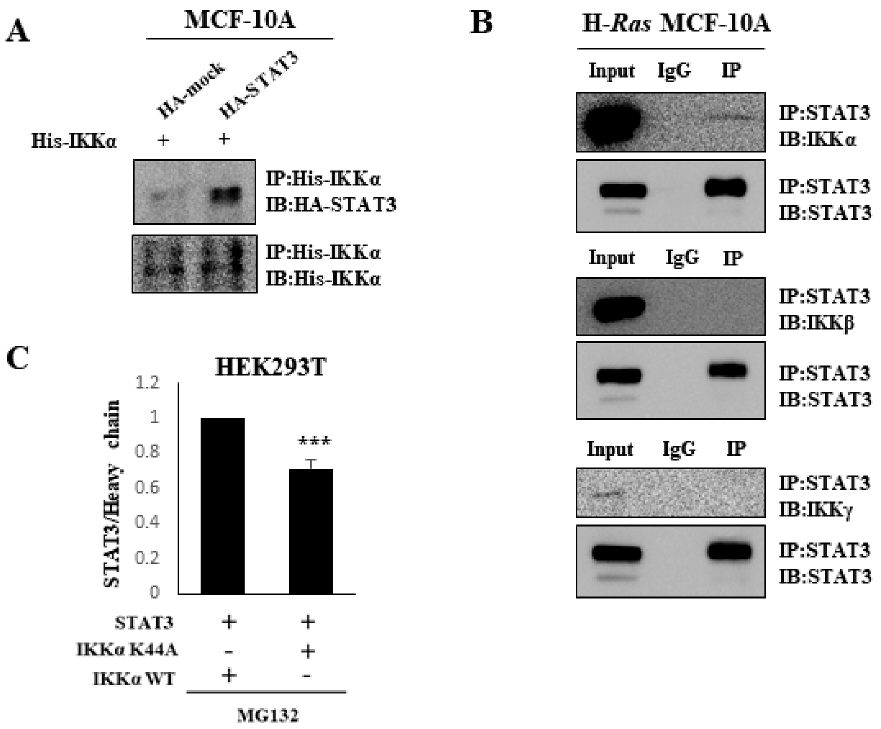

Figure S4. STAT3 is not able to interact with other IKK subunits (A) MCF-10A cells were transfected with mock or STAT3-overexpressing vectors. IP was performed with an anti- IKKα antibody. IB, with IKKα and STAT3 antibodies. **(B)** IP analysis of the interaction between STAT3 and IKK subunits in H-Ras MCF10A cells. IP, with an anti-STAT3 antibody; IB, with antibodies against STAT3 and IKKα, β or γ. **(C)** The densitometry calculations for IP data shown in Figure 5F. The band intensities were normalized by the heavy chain level and presented as bar graphs. ***$p < 0.001$.

a

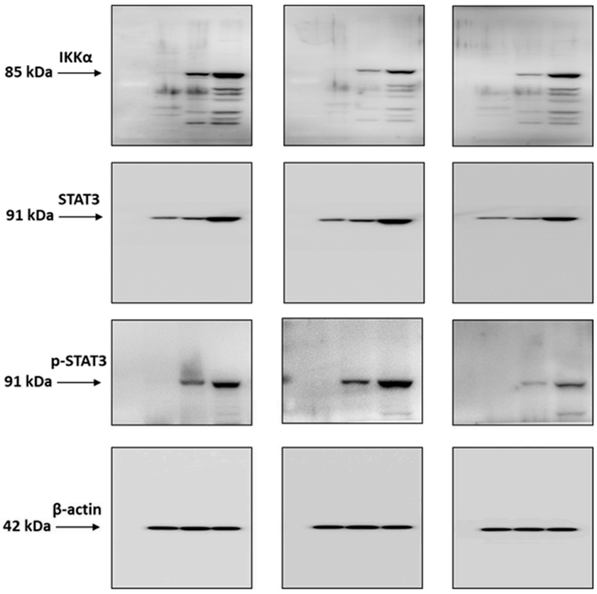

b

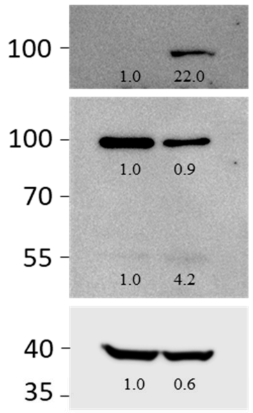

c

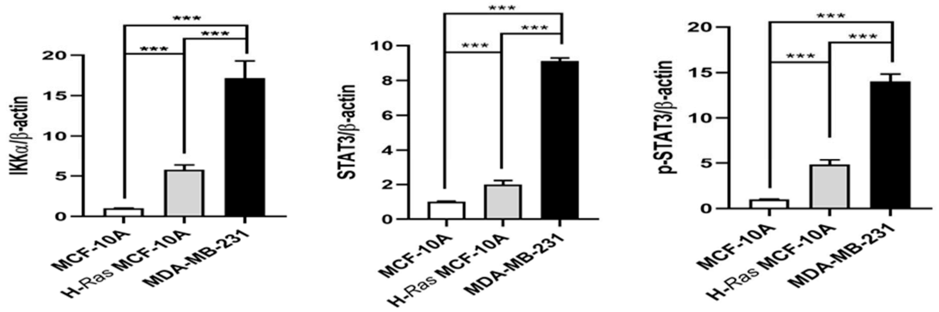

d

b	MCF10A	H-Ras MCF10A
IKKα/Actin	1.00	36.67
p100/Actin	1.00	1.50
p52/Actin	1.00	7.00

Figure S5. The original blot images of Figure 1 (a, b) Full blots corresponding to Fig. 1C and 1F, respectively. (c, d) The band intensity of blots in Fig. 1C and 1F was analyzed by the densitometry readings/intensity ratio using GelPro31 and were normalized to the corresponding Actin value.

a

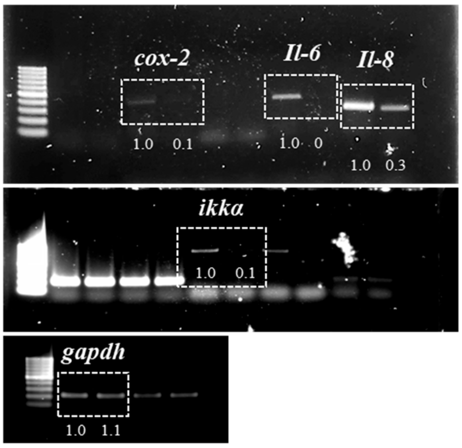

b

a	NC	IKKα siRNA
ikkα/gapdh	1.00	0.09
cox-2/gapdh	1.00	0.09
Il-6/gapdh	1.00	0.00
Il-8/gapdh	1.00	0.27

Figure S6. The original blot images of Figure 2 (a) Full length PCR bands and blots corresponding to Fig. 2A. (b) The band intensity of PCR bands and blots in Fig. 2A was analyzed by the densitometry readings/intensity ratio using GelPro31 and was normalized to the corresponding GAPDH or Actin value.

a **b**

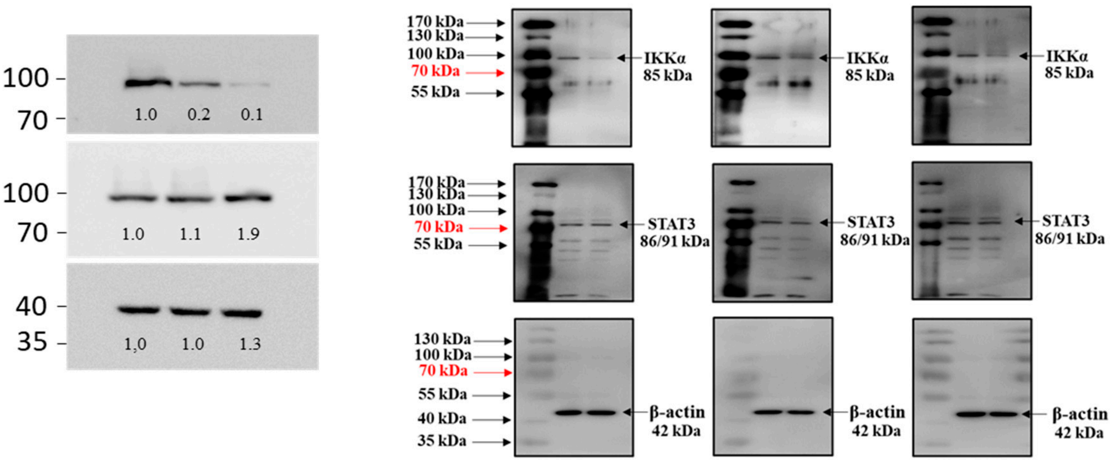

c

a	NC	IKKa siRNA 5 nM	IKKa siRNA 20 nM
IKKα/Actin	1.00	0.20	0.08
STAT3/Actin	1.00	1.10	1.46

d

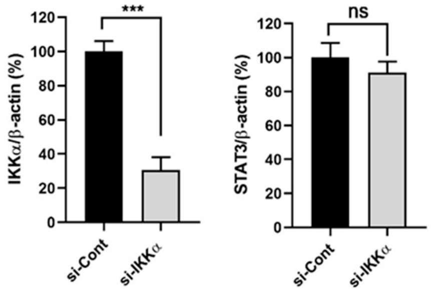

Figure S7. The original blot images of Figures 3A and 3B (a, b) Full blots corresponding to Fig. 3A and 3B respectively. (c, d) The band intensity of blots in Fig. 3A and 3B was analyzed by the densitometry readings/intensity ratio using GelPro31 and was normalized to the corresponding Actin value.

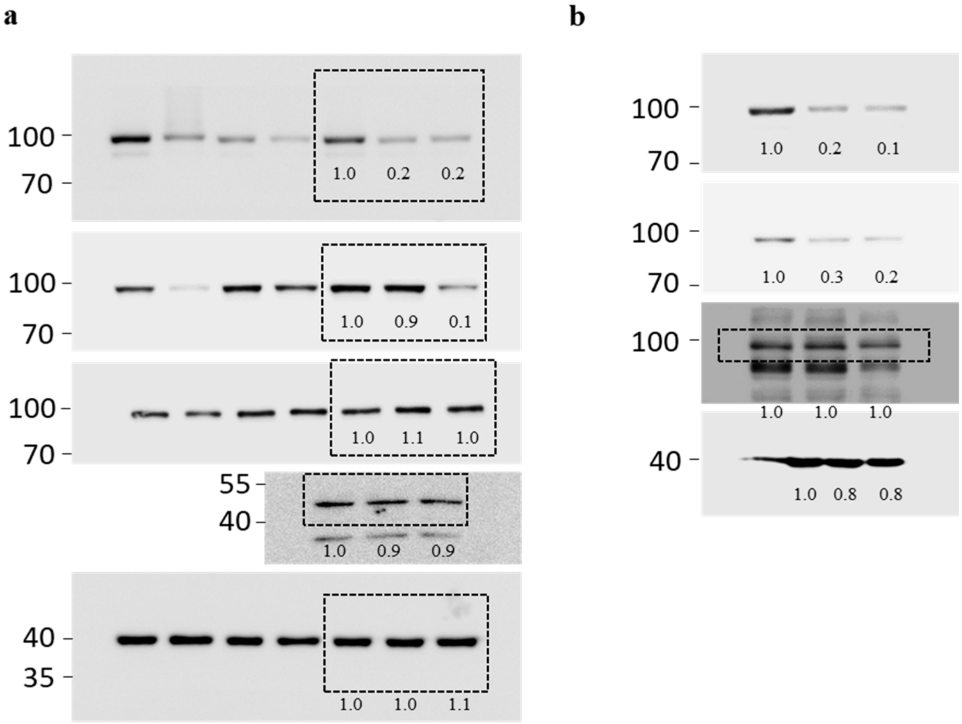

c

b	NC	STAT3 siRNA 5 nM	STAT3 siRNA 20 nM
STAT3/Actin	1.00	0.20	0.18
IKKα/Actin	1.00	0.90	0.09
IKKβ/Actin	1.00	1.10	0.91
IKKγ/Actin	1.00	0.90	0.82

d

b	NC	STAT3 siRNA 5 nM	STAT3 siRNA 20 nM
STAT3/Actin	1.00	0.25	0.13
IKKα/Actin	1.00	0.37	0.25
IKKβ/Actin	1.00	1.25	1.25

Figure S8. The original blot images of Figures 3C and 3D (a, b) Full blots corresponding to Fig. 3C and 3D respectively. (c, d) The band intensity of blots in Fig. 3C and 3D was analyzed by the densitometry readings/intensity ratio using GelPro31 and was normalized to the corresponding Actin value.

a

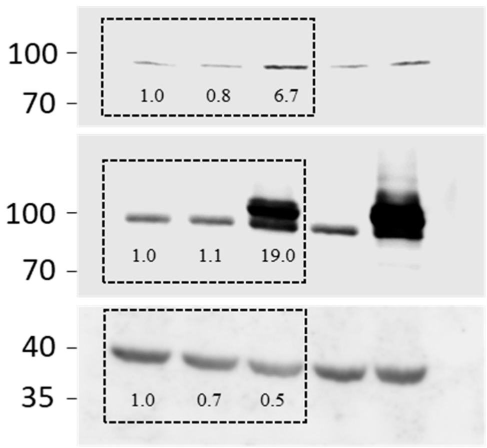

b

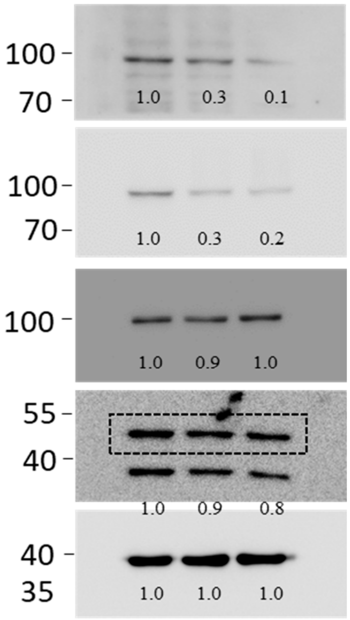

c

	untransfected	mock	WT STAT3
IKKα/Actin	1.00	1.14	13.40
STAT3/Actin	1.00	1.57	38.00

d

	DMSO	Stattic 6 h	Stattic 12 h
pSTAT3/Actin	1.00	0.30	0.10
IKKα/Actin	1.00	0.30	0.20
IKKβ/Actin	1.00	0.90	1.00
IKKγ/Actin	1.00	0.90	0.80

Figure S9. The original blot images of Figures 3E and 3F (a, b) Full blots corresponding to Fig. 3E and 3F, respectively. (c, d) The band intensity of blots in Fig. 3E and 3F was analyzed by the densitometry readings/intensity ratio using GelPro31 and was normalized to the corresponding Actin value.

a

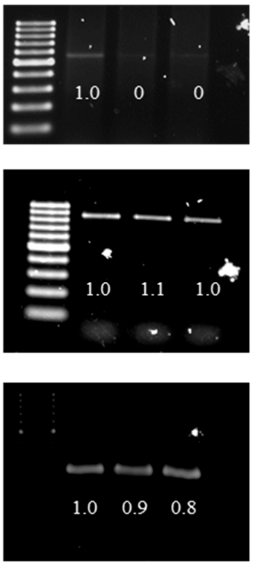

b

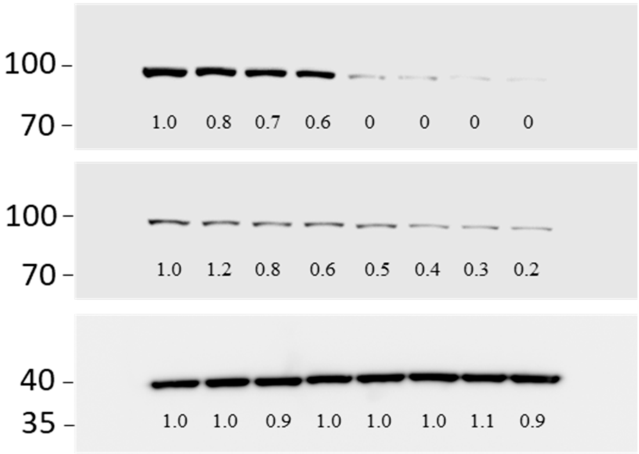

c

a	NC	STAT3 siRNA 5 nM	STAT3 siRNA 20 nM
STAT3/Actin	1.00	0.00	0.00
IKKα/Actin	1.00	0.58	1.25

d

b	NC + CHX 0 h	NC + CHX 0.5 h	NC + CHX 1 h	NC + CHX 3 h	STAT3 siRNA + CHX 0 h	STAT3 siRNA + CHX 0.5 h	STAT3 siRNA + CHX 1 h	STAT3 siRNA + CHX 3 h
STAT3/Actin	1.00	0.67	0.54	0.50	0.00	0.00	0.00	0.00
IKKα/Actin	1.00	1.00	0.89	0.60	0.50	0.40	0.27	0.22

Figure S10. The original blot images of Figures 4A and 4B (a, b) Full bands and blots corresponding to Fig. 4A and 4B respectively. (c, d) The band intensity of bands and blots in Fig. 4A and 4B was analyzed by the densitometry readings/intensity ratio using GelPro31 and was normalized to the corresponding GAPDH or Actin value.

a

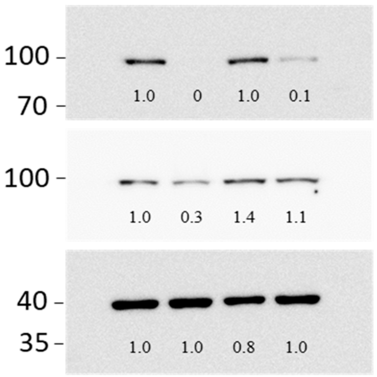

b c

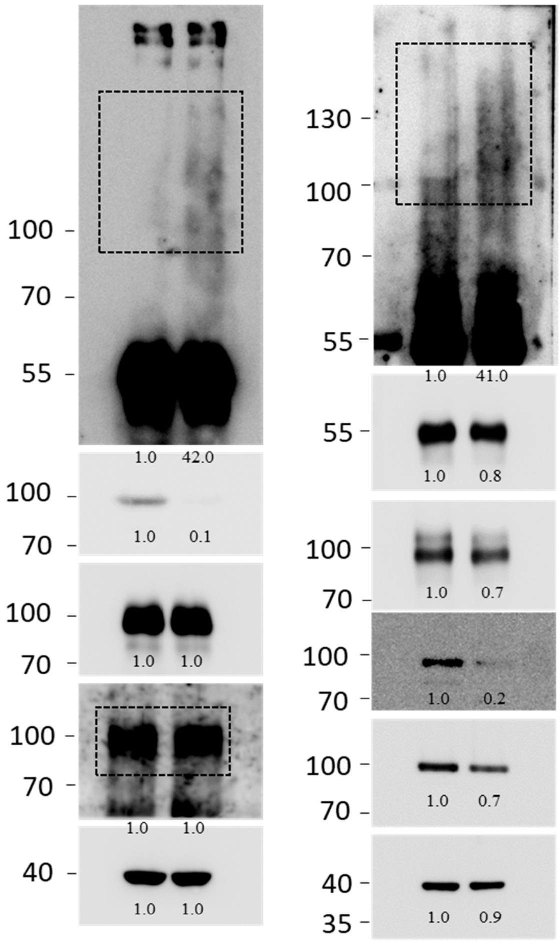

d

c	NC	STAT3 siRNA	NC + MG132	STAT3 siRNA + MG132
STAT3/ Actin	1.00	0.00	1.25	0.10
IKKα/ Actin	1.00	0.30	1.75	1.10

e

d	NC	STAT3 siRNA
STAT3/Actin	1.00	0.10
IKKα + ubquitin/Actin	1.00	42.00
IKKα + ubquitin/ IP: IKKα IB:IKKα	1.00	42.00
IKKα + ubquitin/IgG	1.00	42.00

f

e	NC	Stattic
pSTAT3/Actin	1.00	0.22
STAT3/Actin	1.00	0.78
IKKα + ubquitin/Actin	1.00	45.56
IKKα + ubquitin/IP: IKKα IB:IKKα	1.00	58.57
IKKα + ubquitin/IgG	1.00	51.25

Figure S11. The original blot images of Figures 4C and 4D (a, b, c) Full blots corresponding to Fig. 4C, 4D and 4E respectively. (d, e, f) The band intensity of blots in Fig. 4C and 4D and 4E was analyzed by the densitometry readings/intensity ratio using GelPro31 and was normalized to the corresponding Actin, IgG or IP:IKK IB:IKK*α* value.

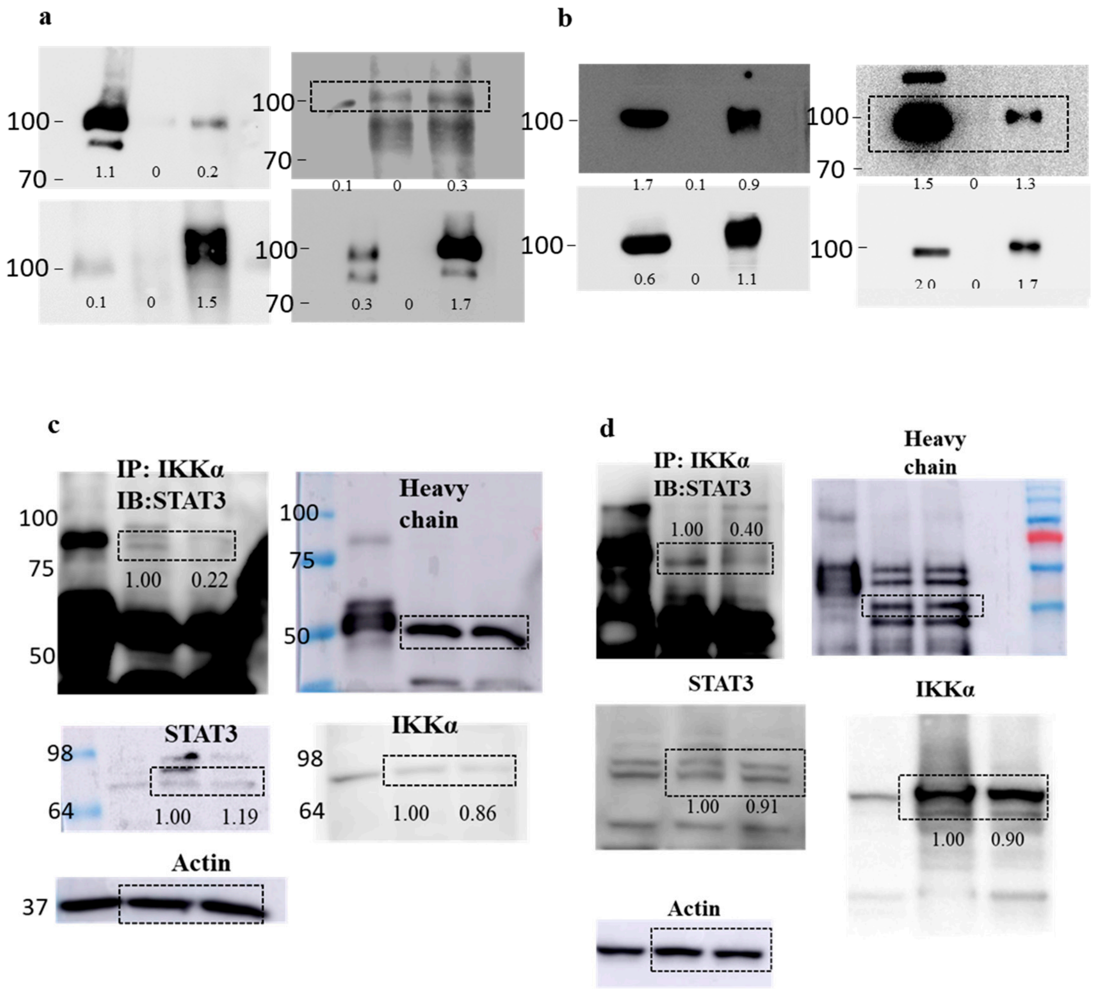

e

	IKKα WT	IKKα K44A
IP: IKKα / heavy chain	1.00	0.22
STAT3 / Actin	1.00	1.19
IKKα / Actin	1.00	0.86

f

	IKKα WT	IKKα K44A
IP: IKKα / heavy chain	1.00	0.40
STAT3 / Actin	1.00	0.91
IKKα / Actin	1.00	0.90

Figure S12. The original blot images of Figure 5 (a, b, c, d) Full blots corresponding to Fig. 5A, 5C, 5E and 5F respectively. (e, f) The band intensity of blots in Fig. 5E and F were analyzed by the densitometry readings/intensity ratio and was normalized to the corresponding Actin value.

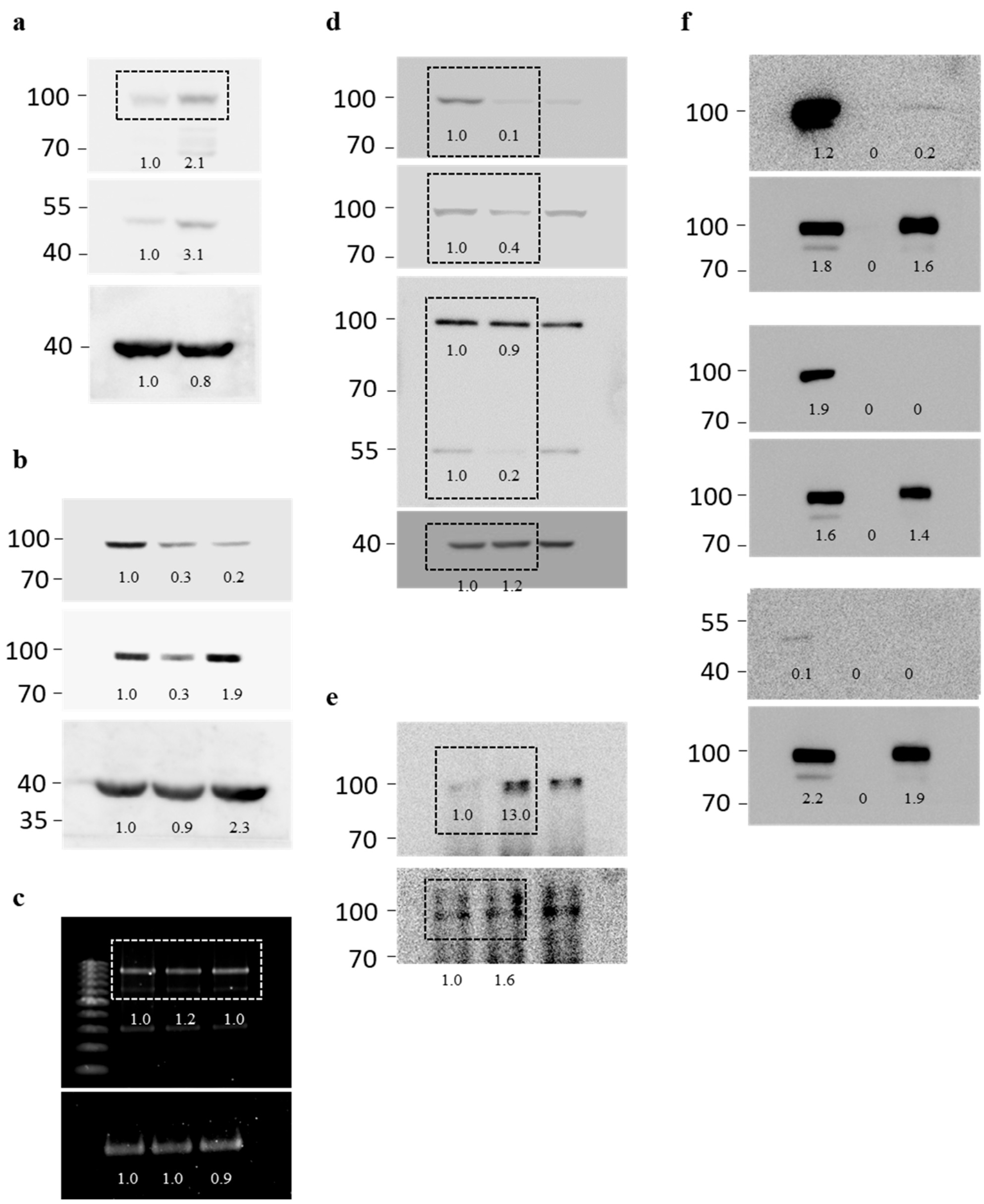

Figure S13. The original blot images of Figures S1 and S3 (a) Full blots corresponding to Fig. S1B. (b, c, d) Full blots corresponding to Fig. S3A, S3C and S3D respectively. (e, f) Full blot corresponding to Fig. S4A and S4B. The band intensity of blots was analyzed by the densitometry readings/intensity ratio using GelPro31 and was normalized to the corresponding Actin or GAPDH.
